# Membrane Interactions of α-Synuclein Revealed by Multiscale Molecular Dynamics Simulations, Markov State Models, and NMR

**DOI:** 10.1021/acs.jpcb.1c01281

**Published:** 2021-03-15

**Authors:** Sarah-Beth T. A. Amos, Thomas C. Schwarz, Jiye Shi, Benjamin P. Cossins, Terry S. Baker, Richard J. Taylor, Robert Konrat, Mark S. P. Sansom

**Affiliations:** † Department of Biochemistry, University of Oxford, South Parks Road, Oxford OX1 3QU, U.K.; ‡ Department of Structural and Computational Biology, Max Perutz Laboratories, University of Vienna, Campus Vienna Biocenter 5, Vienna A-1030, Austria; § UCB Pharma, 208 Bath Road, Slough SL1 3WE, U.K.

## Abstract

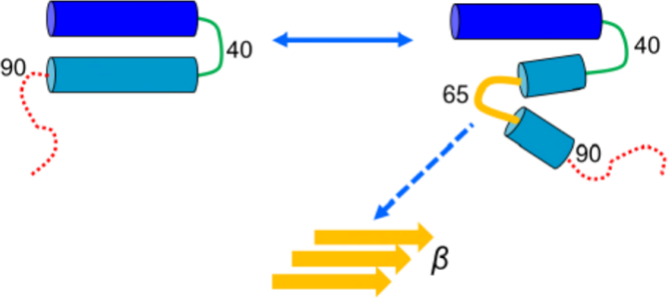

α-Synuclein (αS) is a presynaptic protein that binds to cell membranes and is linked to Parkinson’s disease (PD). Binding of αS to membranes is a likely first step in the molecular pathophysiology of PD. The αS molecule can adopt multiple conformations, being largely disordered in water, adopting a β-sheet conformation when present in amyloid fibrils, and forming a dynamic multiplicity of α-helical conformations when bound to lipid bilayers and related membrane-mimetic surfaces. Multiscale molecular dynamics simulations in conjunction with nuclear magnetic resonance (NMR) and cross-linking mass spectrometry (XLMS) measurements are used to explore the interactions of αS with an anionic lipid bilayer. The simulations and NMR measurements together reveal a break in the helical structure of the central non-amyloid-β component (NAC) region of αS in the vicinity of residues 65–70, which may facilitate subsequent oligomer formation. Coarse-grained simulations of αS starting from the structure of αS when bound to a detergent micelle reveal the overall pattern of protein contacts to anionic lipid bilayers, while subsequent all-atom simulations provide details of conformational changes upon membrane binding. In particular, simulations and NMR data for liposome-bound αS indicate incipient β-strand formation in the NAC region, which is supported by intramolecular contacts seen *via* XLMS and simulations. Markov state models based on the all-atom simulations suggest a mechanism of conformational change of membrane-bound αS *via* a dynamic helix break in the region of residue 65 in the NAC region. The emergent dynamic model of membrane-interacting αS advances our understanding of the mechanism of PD, potentially aiding the design of novel therapeutic approaches.

## Introduction

α-Synuclein (αS) is a protein implicated in neurodegenerative disorders including Parkinson’s disease and Lewy body dementia.^1^ Its function in healthy neurons remains uncertain.^2^ Lipid bilayer association of αS is thought to be important for its biological function in regulating synaptic vesicles, where it has been shown to be essential for SNARE complex assembly at the presynaptic membrane.^3,4^ It has been postulated to have a wide range of functions, including neuronal differentiation^4^ and suppression of apoptosis.^5^ It is thought that toxicity toward neurons arises from the interaction of misfolded/aggregated αS with the lipid bilayer component of cell membranes.^6,7^ Thus, defining the possible modes of interaction between αS and lipid bilayers is a key step in understanding the mode of action of αS, and in the longer term, helping provide a route toward drug research aimed at preventing or reversing its cellular effects.

**Figure 1:**
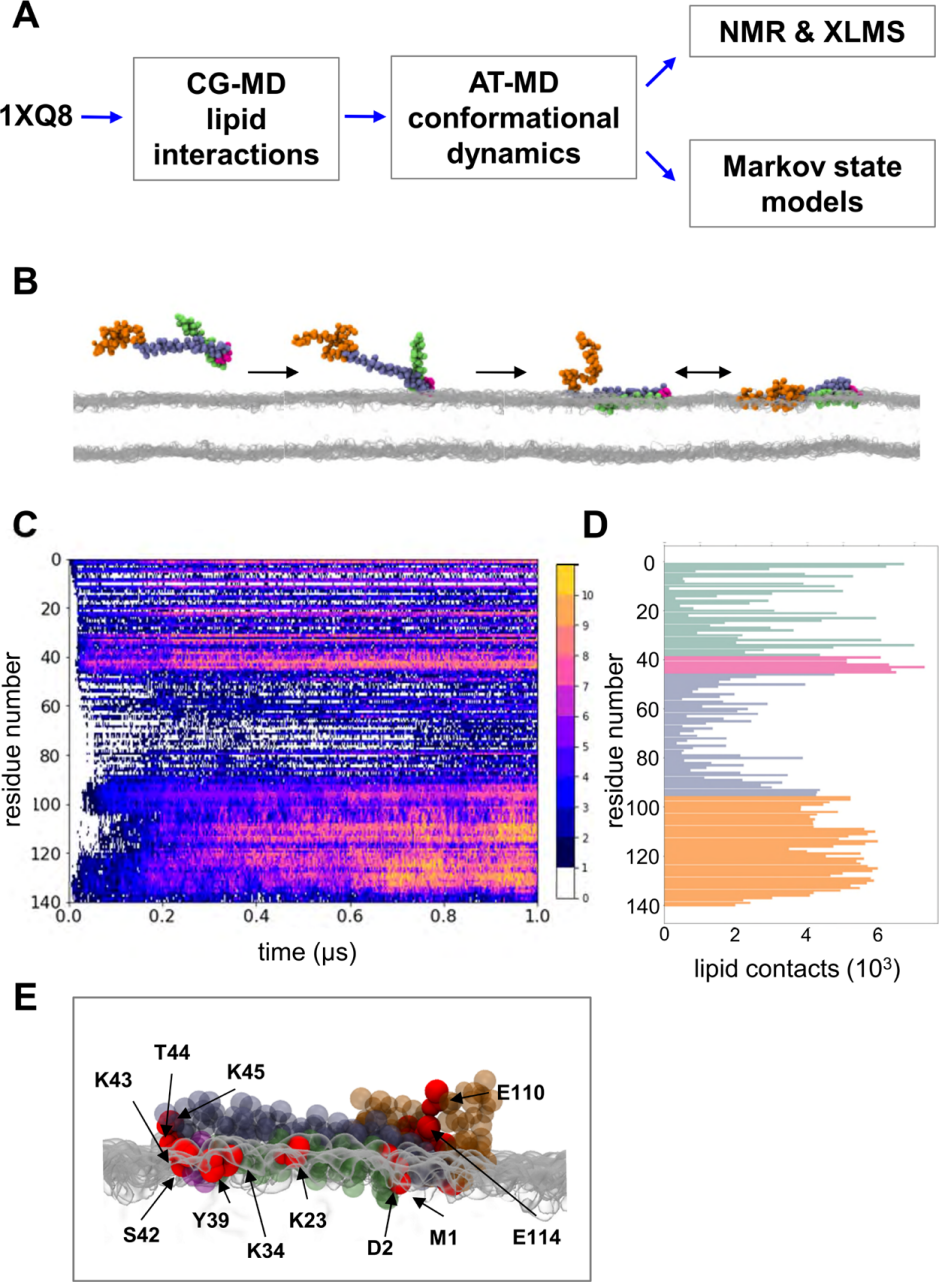
Interaction of αS with a lipid bilayer explored by multiscale simulations alongside biophysical measurements. (A) Flow diagram of the use of CG and AT MD simulations combined with biophysical measurements and MSMs of the AT simulations. (B) Successive snapshots from a CG simulation of the interaction of αS with a PG bilayer. Monomeric αS is initially positioned distal to the membrane. During 10 replicate simulations, each of the 1 μs duration αS bound to the membrane. Colors are based on the initial starting model (PDB id 1XQ8): two helices (helix 1 in green and helix 2 in gray/purple) separated by an interhelical loop (in pink) and followed by a C-terminal disordered region (in orange). (C) Total number of simulations across the ensemble where each residue of αS is in contact with the membrane. (D) Total number of individual contacts of αS with PG summed over the 10 simulation replicates. A residue is considered in contact with the lipid if the residue backbone particle is within 0.7 nm of any lipid particle. (E) Side view of αS bound to the surface of a PG membrane from the endpoint of a simulation. Red particles indicate the top 14 residues making contact with PG lipid headgroups shown in gray.

Native αS is an intrinsically disordered monomeric protein when in aqueous solution.^8−10^ Small-angle X-ray scattering (SAXS) and nuclear magnetic resonance (NMR)-derived experimental data suggest relatively small amounts of compaction in the ensemble of free αS^11,12^ although some discrete molecular dynamics (MD) simulations^13^ combined with cross-linking data^14^ have been interpreted as suggesting that part of the ensemble of structures present may be relatively compact. The partial secondary structure in the free state of αS is observed experimentally in solid-state NMR measurements.^15^ A wide range of studies (recently reviewed by^16^), including solution and solid-state NMR, electron paramagnetic resonance (EPR), FRET, and circular dichroism reveal that αS folds into a predominantly α-helical structure when it interacts with a lipid bilayer (*e.g.*, liposome) or membrane mimetic (*e.g.*, SDS micelle) surface, which provide models for the anionic lipid bilayer component of cell membranes.^15,17−21^ For example, the NMR structure of αS when bound to SDS micelles (PDB id 1XQ8; Figure 1A)^19,22^ is formed by two α-helices (residues 3–37 and residues 45–95) in the N-terminal section of the molecule followed by a disordered C-terminal segment (residues 98–140). Similarly, when bound to sodium lauroyl sarcosinate micelles (PDB id 2KKW), residues 2–32 and 42–92 are α-helical.^23^ A combination of NMR approaches suggests that when αS is bound to anionic lipid bilayers (in small unilamellar vesicles), residues 6–25 form a well-defined membrane-bound α-helix, while residues 26–97 are more conformationally mobile, transiently adopting an α-helical conformation while bound to the membrane surface.^24^ EPR and DEER^25^ studies of αS bound to anionic lipid [phosphatidylcholine (PC)/phosphatidylserine (PS)/phosphatidylglycerol (PG)] vesicles suggested that an extended α-helix is formed from residues 12 to −62 (or beyond) and that the break in the helix in the earlier NMR structures could be due to the curvature of SDS micelles. However, a number of subsequent studies^26−28^ indicated that there may be an equilibrium between extended and broken helix populations and that the broken helix conformation may be adopted by membrane-bound αS. Deep mutational scanning of αS expressed in yeast suggests a membrane-bound α-helical conformation, which increases in dynamics toward the C terminus of the molecule.^29^ Atomistic (AT) simulations using a membrane-mimetic (PC/PS; HMMM) model^30^ and starting from a lauroyl sarcosinate micelle-bound conformation (PDB id 2KKW) indicated a degree of conformational heterogeneity for membrane-bound αS. It should also be noted the α-helical N-terminal segment of αS contains seven KTKEGV motifs, which are thought to play a role in maintaining an unfolded state in solution^31^ and the disordered C-terminal tail contains 10 glutamate and 5 aspartate residues and so is unlikely to form favorable interactions with an anionic lipid bilayer. Thus, overall, it appears that the N-terminal section of αS may adopt either an extended and/or a dynamically disrupted α-helical conformation when bound to a membrane surface, depending on inter alia the membrane composition and curvature.^16^ Significantly, a recent NMR and infrared study of a peptide in the latter part of the overall α-helical region (αS(71–82)) interacting with anionic membranes suggests that this region is also capable of adopting a β-sheet structure.^32^ This is consistent with a degree of conformational dynamics in this region of the membrane-bound αS molecule.

Exogenously delivered αS appears to be present largely as a disordered monomer in the cytoplasm of mammalian cells.^10^ More recently, the anionic lipid phosphatidylinositol phosphate (PIP_2_) has been implicated in localizing αS to the plasma membrane.^33^ Disease-causing mutations alter lipid-induced generation of fibrils.^34^ Mutations of the gene for glucocerebrosidase, which catalyzes hydrolysis of the lipid glucosylceramide, are a major genetic risk factor for Parkinson’s disease.^35^ There is some evidence that oligomeric αS intermediates, which form only on the membrane, are more toxic than the mature fibrils.^36−39^

Computational approaches, and in particular MD, have played a key role in exploring the conformational dynamics of αS and related proteins both in aqueous solution^40−44^ and when bound to membranes.^45−51^ MD simulations have also been shown to be a powerful tool for characterizing the interactions of membrane proteins with lipids^52,53^ and with other molecular surfaces. Recently, for example, MD simulations alongside experimental investigations of αS interacting with nano-objects with different surface properties suggest that charged nano-objects can modulate the fibrillation process.^54^ Exhaustive mapping of the free energy landscape for αS in aqueous solution suggests two main free energy minima, one corresponding to a largely elongated α-helix and the other to a more compact state containing multiple shorter α-helices.^55^

In this context, it is important to characterize interactions of αS with anionic lipid bilayers alongside dynamic conformational rearrangements that may take place while αS is at the membrane surface. Understanding the possible conformations of αS while interacting with a model of a cell membrane is relevant to its pathophysiological role as the modulation of membrane binding as a therapeutic strategy remains a possibility.^56^ Here, we use a multiscale MD simulation approach combining coarse-grained and AT simulations to explore the interaction of αS with anionic lipid bilayers. Markov state model (MSM) analysis of the AT simulations reveals the dynamic behavior of a key element of the α-helical region of the membrane-bound protein.

## Methods

### Computational

The NMR structure of micelle-bound αS^19^ (PDB id 1XQ8) was used and converted to a coarse-grained (CG) representation. CG simulations of αS were performed using a 10 × 10 nm^2^ area bilayer of palmitoyl oleoyl phosphatidylglycerol (POPG) or a lipid mixture of dioleoyl phosphatidylcholine/dioleoyl phosphatidylethanolamine/dioleoyl phosphatidylserine (DOPC/DOPE/DOPS) in a 2/5/3 ratio (see Table 1). The high fraction of DOPE in the latter lipid mixture was chosen to mimic the lipid composition of synaptic vesicles.^57^ Bilayers were built using INSANE.^58^ The protein molecule was positioned 4 nm away from the bilayer surface. The box was solvated and sodium and chloride ions added to a concentration of ∼0.15 M. CG simulations were performed using GROMACS 5.1.^59,60^ Energy minimization was carried out *via* steepest descent and the system equilibrated for 5 ns with protein backbone particles restrained. Productions simulations were run without restraints for 1 or 2 μs with 10 replicates with different initial velocities (Table 1).

**Table 1:** Summary of Simulations

description	CG/AT^a^	lipids	time (μs)	*N*	total (μs)
αS/bilayer	CG	POPG	1	10	10
αS/bilayer, varying helicity	CG	POPG	1	4 × 10	40
αS/mixed lipid bilayer	CG	DOPC/DOPE/DOPS 2:5:3	2	10	20
conformational changes	AT	POPG	0.1–0.25	3 × 10	5.5

a *N* = number of replicate simulations.

CG simulations were performed using the Martini 2.1 force field^61^ with a 20 fs time step. Particle coordinates were written out every 0.5 ns. Coulombic interactions were shifted to zero between 0 and 1.2 nm. Lennard–Jones interactions were shifted to zero between 0.9 and 1.2 nm. The nearest neighbor list was updated every 10 steps. The Berendsen thermostat^62^ (coupling constant 1 ps) and barostat (coupling constant 1 ps, compressibility 5 × 10^–6^ bar^–1^) were used to maintain temperature at 323 K and pressure at 1 bar. The LINCS algorithm^63^ was used to constrain bond lengths.

AT simulations were performed using the Charmm36 force field^64^ with a 2 fs time step. Atomic coordinates were written out every 20 ps. Lennard–Jones interactions were shifted to zero between 0.9 and 1.2 nm. Long-range electrostatic interactions were treated using the particle-mesh Ewald method (PME)^65^ using default parameters pme-order = 4 and ewald-rtol = 10^–5^, fourierspacing = 0.12. PME was shifted from 0 to 1 nm (40). The nearest neighbor list was updated every 10 steps. A velocity-rescale thermostat^66^ (coupling constant 1 ps) and Parrinello–Rahma^67^ barostat (coupling constant 1 ps, compressibility 5 × 10^–6^ bar^–1^) were used to maintain the temperature and pressure. The LINCS algorithm was used to constrain bond lengths.

AT simulations were started from snapshot structures taken from CG simulation and converted to AT representations.^68^ AT-MD simulations were performed using GROMACS 5.1/2018. The system was equilibrated for 1.5 ns with the backbone atoms of the protein restrained and then a production run was performed (Table 1).

VMD was used for simulation visualization.^69^ Graphs were generated in matplotlib^70^ and seaborn. Contact analysis was carried out with in-house Python scripts using a cutoff of 0.7 nm for CG and 1.0 nm for AT from the Cα carbon atom. Principal component analysis and correlation analysis were carried out using standard libraries in matplotlib/seaborn (seaborn.pydata.org) and scikit-learn.^71^ Markov state modeling was carried out in MSMBuilder.^72^ The system was featurized on backbone contacts and scaled using StandardScaler. The number of microstates was set at 50 for calculation and implied timescales plotted to assess convergence. The system was clustered into macrostates (this is further explained in the context of the results) and the two lowest energy states were used to extract representative structures which were visualized with VMD.

## Experimental Section

### Nuclear Magnetic Resonance

αS was expressed and purified as described previously.^73^ Secondary shift propensity scores (SSPs) were calculated from H, HN, CO, Cα, and Cβ shift values obtained in phosphate buffer pH = 5.5, 323 K. They were calculated using the SSP-script^74^ using Cα and Cβ to apply internal referencing. Assignments of αS in these conditions were obtained using TROSY^75^ versions of 3D backbone assignment pulse sequences for the bicelle bound form of αS. In order to obtain sufficient signal intensity, the protein was partially deuterated by expression in D_2_O-based M9 media. Bicelles were composed of DHPC (dihexanoyl phosphatidylcholine), DMPG (dimyristoyl phosphatidylglycerol), and PIP_2_ with final concentrations of 100 μM αS, 10.6 mM DHPC, 5.1 mM DMPG, and 1.1 mM PIP_2_; all bicelle components were sourced from Avanti Polar Lipids. According to Glover *et al.,*(76) a significant fraction of DHPC is expected not to incorporate into bicelles; therefore, these bicelles are expected to contain about 3.6 mM DHPC. The ^15^N relaxation of micelle-bound αS was measured using 150 μM protein and 40 mM SDS (Sigma-Aldrich) concentrations in phosphate buffer pH = 5.5, 323 K at a field corresponding to a ^1^H Lamor frequency of 599.89 Hz. We employed Carr–Purcell–Meiboom–Gill (CPMG) delays of 16, 34, 68, 136, 204, 271, 407, and 543 ms using a duty cycle of 0.5 ms to determine the ^15^N transverse relaxation rate. Processing of the spectra and fitting of exponential decay curves were carried out using the software SPARKY.^77^

### Cross-linking Mass Spectrometry

Cross-linking of αS was carried out with the zero-length 1-ethyl-3-(3-dimethylaminopropyl)carbodiimide (EDC, Sigma-Aldrich) in the presence of liposomes composed of POPG (Avanti Polar Lipids) in phosphate buffer pH = 6.5. 50 μM of αS was incubated for 60 min at room temperature in the dark with 2 mM EDC and 5 mM hydroxy-2,5-dioxopyrrolidine-3-sulfonic acid (Sulfo-NHS, Sigma-Aldrich) with 1 mg/mL of POPG-based liposomes present during the reaction. Liposomes were extruded through 0.4 μM filter membranes (Avanti Polar Lipids) and a regular size distribution was verified by dynamic light scattering (DynaPro NanoStar, by Wyatt). Under these conditions, the majority of αS is bound to the liposomal surface.^73^ The reaction was stopped with Tris (50 mM) and β-mercaptoethanol (20 mM). The resulting cross-linked protein was then subjected to SDS-PAGE, which showed monomer, dimer, and multimer bands of αS. The band corresponding to the monomeric weight of αS was selected for further analysis in order to avoid interference by cross-links stemming from intermolecular interactions. Liposomes used in this reaction were produced as described previously.^73^

The monomer band was excised from the gel and destained with a mixture of acetonitrile (Chromasolv, Sigma-Aldrich) and 50 mM ammonium bicarbonate (Sigma-Aldrich). The proteins were reduced using 10 mM dithiothreitol (Roche) and alkylated with 50 mM iodoacetamide. Trypsin (Promega; Trypsin Gold, Mass Spectrometry Grade) and chymotrypsin (Promega sequencing grade) were used for proteolytic cleavage. Digestion was carried out with trypsin at 37 °C overnight and subsequently with chymotrypsin at 25 °C for 5 h. Formic acid was used to stop the digestion and extracted peptides were desalted using C18 Stagetips.^78^

Peptides were analyzed on an UltiMate 3000 HPLC RSLCnano system (Thermo Fisher Scientific) coupled to a Q Exactive HF mass spectrometer (Thermo Fisher Scientific) equipped with a Nanospray Flex ion source (Thermo Fisher Scientific). The samples were loaded on a trap column (Thermo Fisher Scientific, PepMap C18, 5 mm × 300 μm ID, 5 μm particles, 100 Å pore size) at a flow rate of 25 μL min^–1^ using 0.1% trifluoroacetyl as the mobile phase. After 10 min, the trap column was switched in-line with the analytical C18 column (Thermo Fisher Scientific, PepMap C18, 500 mm × 75 μm ID, 2 μm, 100 Å) and peptides were eluted applying a segmented linear gradient from 2 to 80% solvent B (80% acetonitrile, 0.1% formic acid; solvent A 0.1% formic acid) at a flow rate of 230 nL/min over 120 min. The mass spectrometer was operated in data-dependent mode, survey scans were obtained in a mass range of 350–1650 *m*/*z* with lock mass activated, at a resolution of 120,000 at 200 *m*/*z* and an AGC target value of 3 × 10^6^. The 10 most intense ions were selected with an isolation width of 1.6 Thomson for a max. of 250 ms, fragmented in the HCD cell at 28% collision energy and the spectra recorded at a target value of 1 × 10^4^ and a resolution of 60,000. Peptides with a charge of +1, +2 or >+7 were excluded from fragmentation, the peptide match feature was set to preferred, the exclude isotope feature was enabled, and selected precursors were dynamically excluded from repeated sampling for 20 s within a mass tolerance of 8 ppm.

For peptide and protein identification, raw data were processed using the MaxQuant software package^79^ (version 1.5.5.1) and spectra searched against a combined database of the αS construct sequence, the *Escherichia coli* K12 reference proteome (UniProt), and a database containing common contaminants. The search was performed with full trypsin and chymotrypsin specificity and a maximum of three missed cleavages at a protein and peptide spectrum match (PSM) false discovery rate of 1%. Carbamidomethylation of cysteine residues was set as fixed and oxidation of methionine and N-terminal acetylation as variable modifications. All other parameters were left at default. To identify cross-linked peptides, the spectra were searched using pLink^80^ (version 1.23). Q Exactive HF raw-files were pre-processed and converted to mgf-files using pParse.^81^ The spectra were searched against a database containing the eight most abundant protein hits [sorted by MS/MS counts] identified in the MaxQuant search. Carbamidomethylation of cysteine and oxidation of methionine residues were set as variable modifications. Trypsin/chymotrypsin was set as enzyme specificity, EDC was set as cross-linking chemistry allowing Asp and Glu residues to be linked to Lys residues. Search results were filtered for 1% FDR at the PSM level and a maximum allowed precursor mass deviation of 5 ppm. To remove low quality PSMs, an additional e-Value cutoff of <0.001 was applied. Proteomics analyses were performed by the Max Perutz Laboratories Mass Spectrometry Facility using the VBCF instrument pool.

The resulting pattern of cross-links is very varied due to the high flexibility of bound αS. Looplinks were not included in this analysis as these would show short range contacts only. We show the resulting crosslink-pattern in terms of PSMs plotted with binwidth = 1 (in Figure 5A below) and as a density plot generated with the 2D kernel density estimation using stat_density2d in R with parameter geom = “tile” generated with the R-package ggplot2^82^ (in the corresponding Supporting Information Figure S5A).

## Results

In order to investigate the interactions of αS with model membranes, we adopted a multiscale approach combining CG simulations of the protein/bilayer encounter followed by AT simulations to explore conformational changes of the protein when bound to the bilayer surface. The results from the AT simulations are compared with biophysical [NMR and cross-linking mass spectrometry (XLMS)] data and are analyzed using an MSM (see Figure 1A and Table 1 for a summary of the simulations performed).

### CG Simulations Reveal the Initial Membrane Binding Mode of αS

CG simulations were set up by positioning an αS monomer (in a CG representation) corresponding to a structure in the presence of SDS micelles (PDB id 1XQ8; see Supporting Information Figure S1A) 4 nm away from the surface of an anionic POPG bilayer (Figure 1B). This structure was selected to represent a “broken-helix” conformation as has been observed for both membrane-bound and micelle-bound αS (see above and a recent review^16^). As can be seen from Figure 1B, the CG simulation (which includes restraints to maintain the initial secondary structure of the protein) allows considerable flexibility of the αS molecule in both the disordered C-terminus (in orange in Figure 1B) and around the helix break close to residue 40 (pink in Figure 1B), while the two α-helical regions (in green and gray/purple in Figure 1B) are able to move relative to one another and to the bilayer. The protein molecule diffused toward and bound to the membrane surface within less than 1 μs. These simulations revealed that the end of the first helix (around residue 35) and the following interhelical break region formed the first contacts with the POPG membrane (Figure 1B,C). In all simulations, the protein bound to the bilayer within 1 μs. Residues in the N-terminal helix 1 (residues 1–38) forming multiple contacts with POPG headgroups included residues 1, 21, 23, 32, and 34. In contrast, helix 2 (residue 46–95) formed few direct contacts with the POPG membrane, although residues 50 and 80 contributed to the overall binding profile as the simulation progresses. A representative bound structure of αS with the top 14 residues making lipid contacts highlighted in red is shown in Figure 1E (and in Supporting Information Figure S1B). Overall, we observed that helix 1 formed close contacts with the membrane, with major contributions to the contact-binding profile from residues M1, D2, K23, and K34. This correlates well with the tight membrane binding of this helical region seen in NMR studies.^24^ Interestingly, we observe that helix 2, which contains the NAC (non-amyloid-β component) region of the protein is folded back over the top of helix 1, thus forming fewer direct contacts with the lipid bilayer. The disordered C-terminus makes a number of contacts with the membrane as the simulation progresses (Figure 1C,D). This correlates with experimental observation of calcium-mediated contacts of the C-terminus to synaptic vesicles.^83^

As noted above, in the CG protocol, the secondary structure present in the initial structure is restrained during the simulation, while secondary structure elements are able to move relative to one another. To establish how changes in the initial secondary structure restraints might modulate the subsequent bilayer interactions, we modeled the αS monomer with decreasing degrees of secondary structure restraints in the CG simulations: residues 1–80, 1–60, 1–40, and 1–20 (Table 1 and Supporting Information Figure S2). As the restraints were relaxed, we observed a greater contribution to the bilayer contact profile from the helix 2 region. The increased binding contribution from the helix 2 region reflects its greater conformational freedom to make contacts with the bilayer instead of being folded over helix-1.

**Figure 2:**
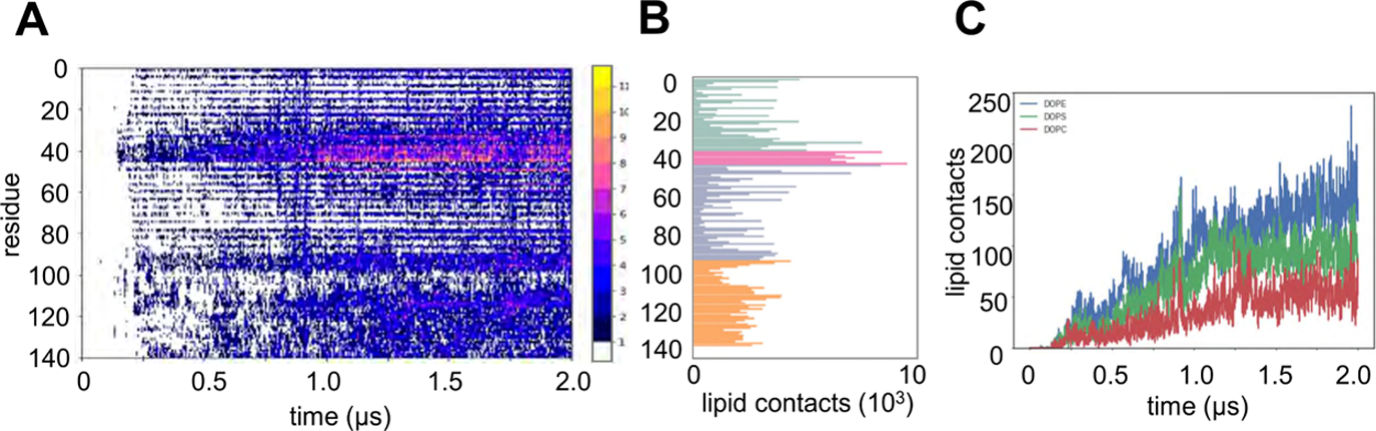
CG MD simulations of the interaction of αS with a PC/PE/PS (2:5:3) lipid bilayer. Simulations were of 10 replicates each for 2 μs. (A) Contacts for all lipids for each residue of αS summed over the 10 simulation replicates. (B) Total contacts shown separately for the three lipid species. Colors on the histogram indicate the structural regions defined in Figure 1B (helix 1 in green, helix 2 in gray/purple, interhelical loop in pink, C-terminal disordered region in orange). (C) Total contacts for each lipid species (PC = red; PE = blue; PS = green) shown as a function of time.

We also explored the binding of the αS monomer to a more complex anionic model membrane. The mixed lipid bilayer employed (DOPE/DOPS/DOPC in a 5:3:2 M ratio) was intended to provide a simple mimic of a synaptic vesicle membrane. This is anionic but with 30% of the net surface charged of the POPG bilayer used previously. As can be seen from Figure 2A,B, the initial interaction remains led by the end of helix 1 and the following “break” region, with a reduced contribution from the unstructured C-terminus. Analyzing the contribution of each lipid species to the contact profile (Figure 2C and Supporting Information Figure S3) shows that interactions with DOPE and DOPS are preferred to those with DOPC, as has been observed for a number of experimental studies^84^ reflecting the H-bonding propensity of the PE and PS headgroups relative to that of PC. Overall, these simulations confirm those with POPG in indicating the importance of helix-1 and the interhelical break region. A number of natural mutations are found in these two regions, which alter the membrane-binding affinity, including A30P^85^ and E46K^86^ both of which are associated with differentiated disease pathology.^34^

### Interactions in AT Simulations Correlate with Experimental Biophysical Observations

**Figure 3:**
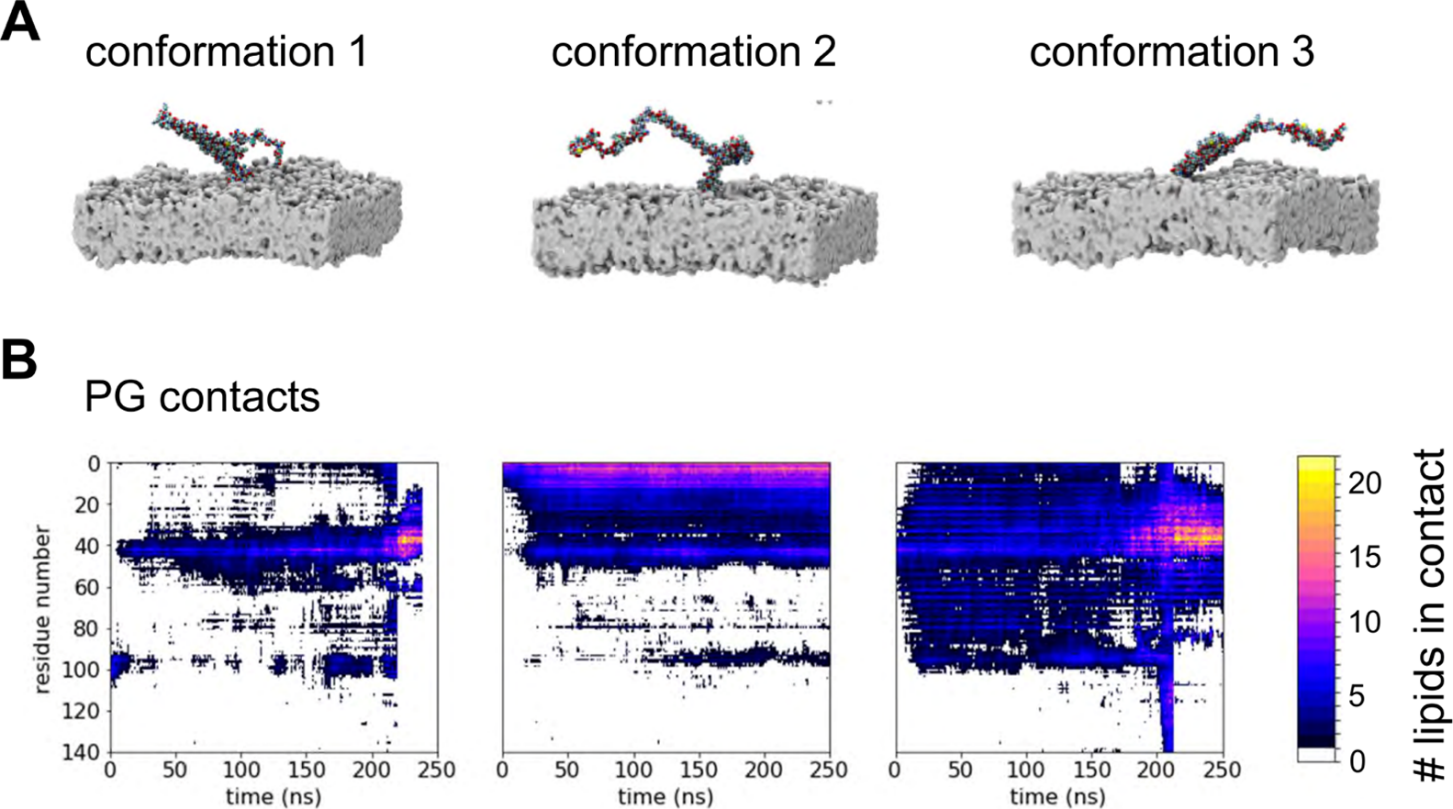
AT MD simulations of the interaction of αS with a POPG membrane. (A) Three binding poses taken from the CG simulations are shown, chosen to capture initial interactions at the C-terminus (conformation 1-left), at the N-terminus (conformation 2-center) and at the inter-helical region (conformation 3-right). In each case, the CG binding pose was converted to the corresponding AT models shown, initiating AT simulations (of duration 100–250 ns with 10 replicates) and resulting in an aggregated simulation time of 5.5 μs. (B) Number of contacts to lipids within a 1 nm cutoff of the Cα atom of each residue is shown as a function of time and residue number for each starting model, averaged across replicates.

The CG simulations enable us to effectively sample protein/membrane encounters and interactions but as noted above, the use of secondary structure restraints reduces sampling of possible conformations of the bound protein. To explore in more detail the membrane interactions starting from protein poses on the bilayer generated by CG simulations, allowing more fully for possible conformational changes, we performed AT simulations starting from instances of αS binding to the membrane taken from the CG/POPG simulations (Figure 3A). Thus, we selected three representative structures of the bound monomer from the CG simulations and converted them to AT resolution.^87^ Each of these three systems formed the starting point for 10 replicates of AT-MD simulations each of duration 0.1–0.25 μs, yielding an aggregated simulation time of 5.5 μs (see Figure 3A and Table 1). Note that these three starting structures represented different initial contacts with the membrane, involving the interhelical break for starting conformation 1, helix 1 for conformation 2, and both helix 1 and helix 2 (and the break in between) for conformation 3. Analysis of the lipid contacts as a function of time (Figure 3B) shows that while both the contact profile and the bilayer penetration are variable across the ensemble of simulations, overall it appears that the αS monomer prefers to bind initially through helix 1 and/or interhelical region, but that there is flexibility in the binding pose such that helix 2 may also form interactions (see especially for starting conformation 3).

**Figure 4:**
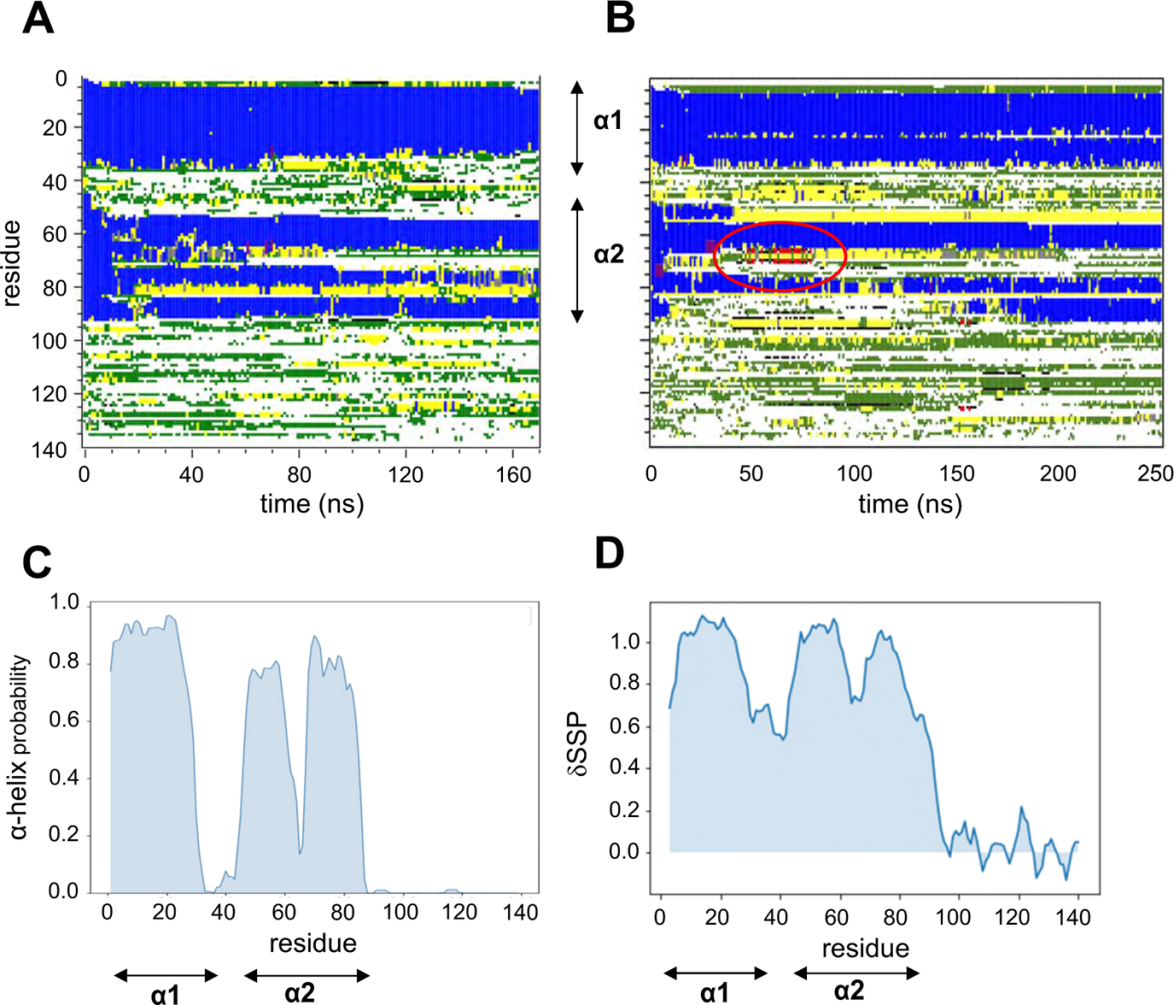
Secondary structure of αS, comparing AT simulations when bound to a POPG bilayer with NMR data. (A) Secondary structure (as defined by DSSP; blue = α-helix; red = β-sheet; green = bend, yellow = turn, white = coil) as a function of residue number and time for a representative trajectory starting from conformation 1 (see Figure 3 above). Loss of helical structure in the middle of helix 2 (in the region of residue 70) is observed. The locations of helices 1 and 2 in the NMR structure (PDB id 1XQ8) are indicated to the right of the diagram. (B) Comparable secondary structure plot for a representative trajectory starting from conformation 2. The red ellipse highlights formation of a small region of β-sheet around residue 70. (C) Frequency of an α-helical secondary structure as a function of residue averaged across all simulations starting from conformation 1. The fraction of α-helix is reduced in favor of random coil conformations between residues 60 and 70. (Similar profiles are seen for simulations starting from conformations 2 and 3). (D) NMR chemical shift data showing secondary structure propensities. Chemical shift indexing shows field-shifted atoms in the region between residues 60 and 75, indicating a reduction in the propensity of the α-helical structure. The values obtained are the average of the shift observed *versus* random coil expected shifts, weighted by their sensitivity to α-helical or extended conformations. Data are shown for αS bound to bicelles composed of DHPC, DMPG, and PIP_2_ (see Methods for details). The extent of the two helices in the SDS-bound structure (PDB id 1XQ8) is again indicated by arrows.

During the course of the AT simulations, we frequently observed a break in the middle of the region corresponding to helix 2 in the initial model. Representative trajectories (Figure 4A,B) show helix 2 (residues 46–95) to be quite dynamic in terms of secondary structure, breaking between residues 65 and 70 from in addition to a degree of expansion of the break around residue 40 present in the initial model. Averaging over 10 replicate simulations (Figure 4C) shows that residues 60–70 have a substantially reduced probability of adopting an α-helical conformation. This profile is in excellent agreement with the equivalent profile derived from NMR chemical shift data for αS bound to anionic phospholipid bicelles (DHPC/DMPG/PIP_2_ ∼3:5:1) (Figure 4D; also see Supporting Information Figure S4A), which show reduced α-helicity in the same region, with values nearly as low as in the initial inter-helical break region around residue 40. The regions which retain a high probability of forming an α-helical structure in the AT simulations match well with the regions of higher α-helical propensity observed in the NMR measurements. The small differences in the profiles may reflect the different lipids employed (POPG *vs* DHPC/DMPG/PIP_2_; interestingly a recent study has suggested αS may interact with PIP_2_ in cell membranes^33^) and also the differences between a lipid bilayer and a bicelle environment, although in both the simulations and the experiments, the membrane surface was predominantly anionic. Overall, these profiles, both experimental and computational, agree well with the suggestion from earlier NMR studies of a tightly bound N-terminal helix and a more transiently interacting subsequent helical region^24^ and align well with a recent model of αS interactions with lipid-bilayer nanodiscs.^88^

Interestingly, in some of the AT simulation trajectories, the β-strand structure is observed in residues in the middle of helix 2 (residues 65–70; see Figure 4B). This suggests that more extended β-structures could be seeded hereabouts in the NAC region, thereby initiating hydrophobic aggregation of αS molecules. This correlates well with, for example, recent observations of β-sheet formation by a peptide (αS71-82) corresponding to this region.^32^

**Figure 5:**
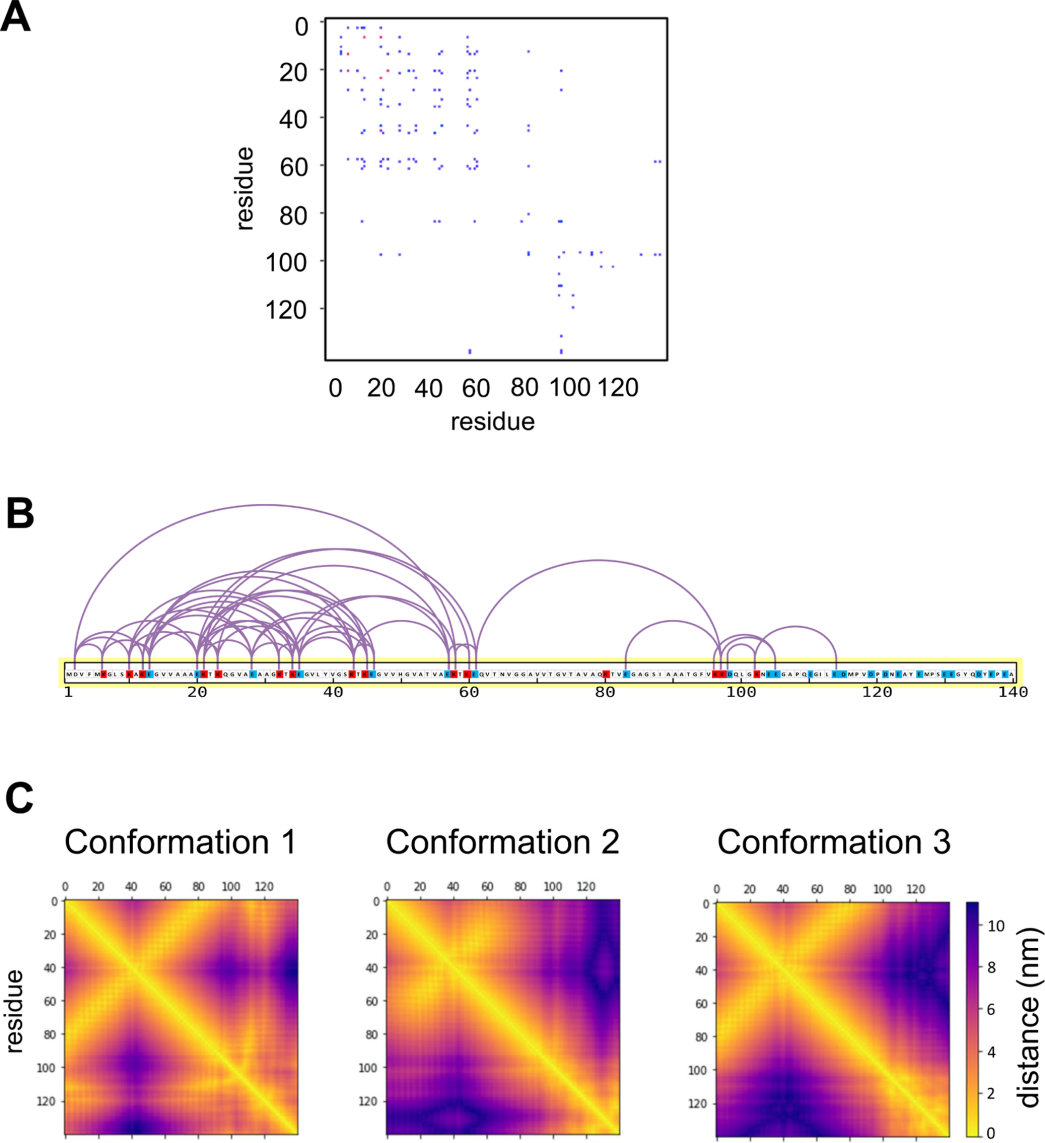
Residue–residue distances of the αS monomer binding to a POPG bilayer, comparing the results of XLMS studies with contacts in simulations. (A) XLMS crosslink-pattern observed in αS when bound to POPG liposomes. The XLMS data are used to determine a sum of all PSMs found between two positions of the protein. This is shown as a pattern of PSMs determined with binwidth = 1, indicating the number (red = higher, blue = lower) of PSMs for each individual cross-link pair. (B) XLMS-pattern mapped onto the sequence of αS with K (blue), E and D (red) residues indicated. (C) Residue–residue distances generated from the AT simulation ensembles from conformations 1, 2, and 3. Off-diagonal elements are seen clearly in both the experimental data and the simulations, corresponding to contacts between helix 1 and the N-terminal segment of helix 2.

We have also compared our simulations of αS at the bilayer surface with experimental data on the larger scale structure of the protein obtained *via* XLMS studies with anionic phospholipid (POPG) liposomes (Figure 5). The large number of different cross-links observed (Figure 5A,B) is as expected for the highly dynamic conformational ensemble of the inosine 5′-diphosphate αS, which retains a large degree of flexibility even in its membrane-bound state.^24^ Sequence mapping of the XLMS-pattern was generated with the program xiNET (Figure 5B).^89^ The XLMS-data shown (Figure 5A) presents the sum of all PSMs found between two positions of the protein as 2D “contact map”. As X-linking positions are restricted to the N-terminus, Lys, Asp, and Glu residues and the amount of PSMs found vary strongly, we also applied a density estimation to the PSM pattern found for easier visual comparison of XLMS and simulation data (see Supporting Information Figure S5). A comparison of these XLMS PSM patterns with intra-monomer contact matrices derived from simulation trajectories (Figure 5C) shows good agreement between experimental measurements and the simulations. This is especially true for the off-diagonal contacts observed between regions 50–70 and 10–30. The full diagonal visible in the simulation data (Figure 5C) is missing from the XLMS picture due to the chemical cross-linker used, the restrictions in places on the amino acids to be linked, and the requirement for the PSMs to stem from two separate peptides. Interestingly, the N-terminal region that shows many PSMs close to the diagonal is dominated by PSMs with an amino acid spacing of 3, 4, or 6 residues fitting with the assumption that they link side-chains within an α-helix. The observed strong diagonal is caused by a higher amount of PSMs at the N-terminus fit with NMR and simulation observations of a stable helix 1 at the N-terminus when the protein is membrane bound.^24,90^

Although it is not as disordered as in solution, αS in equilibrium between water-soluble and membrane-interacting forms retains a high degree of flexibility in its membrane-bound state.^24^ As noted above, this leads to a large number of detected cross-links and their spatial distribution precludes us from evaluating the cross-links found on the basis of a single structure. Recently, studies on the solution state of αS have used a large dataset generated with different cross-linkers as the basis for constraint-guided discrete MD in order to calculate an ensemble of structures fulfilling XLMS-derived distance restraints.^13^ Although the membrane-bound state of αS is less flexible, both the lower amount of distance information available in our case as well as the poor matching of NMR- and SAXS-derived ensembles with those generated using XLMS data in the published structures led us to use a different approach. Here, we show the similarity in interacting regions as derived from experimental and computational approaches. Both of these capture a large ensemble of structures and without explicit calculation of ensembles for the experimental data, we can demonstrate similar behavior as observed in simulations. Intramolecular contacts within membrane-bound αS are analyzed by AT simulations and represented as heat maps (Figure 5C). In all simulations, the strong off-diagonal elements show that the helix 1 and helix 2 regions lie adjacent to each other. As the XLMS data stem from the bound form of the protein, where long contact times lead to strong binding of the N-terminus of the protein,^24^ this observation matches with the observed best agreement between XLMS data and the profile of conformation 2.

### MSMs of the Conformational Changes of Membrane-Bound αS

**Figure 6:**
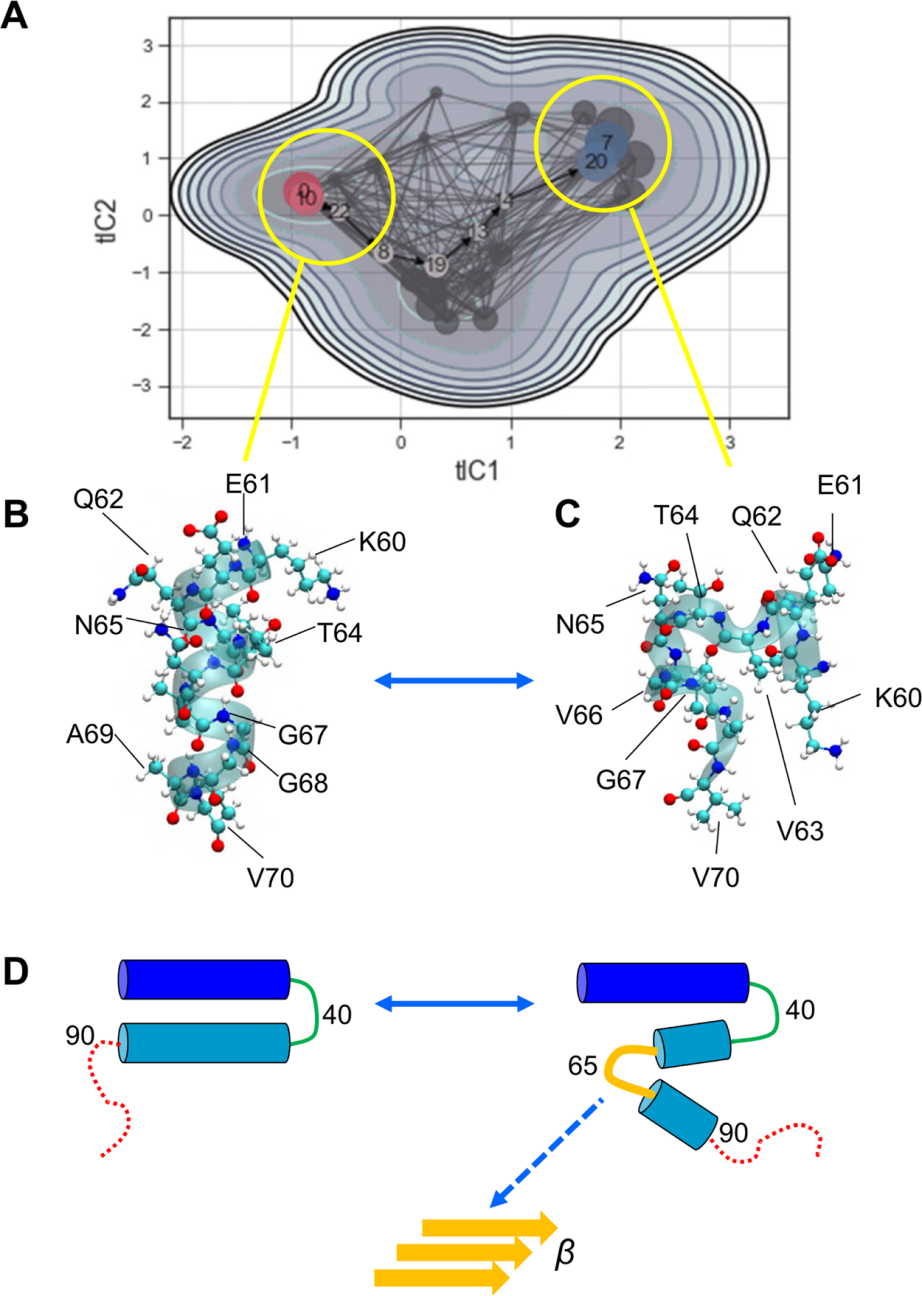
MSM of the conformational dynamics of residues 60–70 based on the AT simulations. (A) MSM microstate transitions and representative structures. Yellow circles indicate the two lowest energy macrostate centers from which representative structures (B,C) were extracted. (B) Residues 60–7 in an α-helical conformation and generated from a region that is structurally close to the conformation at the start of the trajectory. (C) Break in the helix such that the peptide backbone around residues 60–70 folds back on itself. (D) Schematic representation of the dynamic structure of αS bound to an anionic membrane showing helix 1 in blue, the interhelix loop around residue 40 in green, helix 2 in cyan, the dynamic break/possible β-sheet region centered around residue ∼65 in yellow, and the C-terminal disordered region in red. The potential seed region for subsequent amyloid formation is indicated by the yellow schematic labeled β.

To further investigate the conformational changes of the αS monomer when bound to a POPG bilayer, we constructed MSMs using MSMBuilder.^72^ In these, we focused on the conformational dynamics of residues 60–70 of the bound αS monomer as our previous analysis (above; Figure 4) had indicated that this is where the break in helix 2 occurs. We therefore used the pooled data from the AT-MD simulations to construct an MSM featurized on the Cα contacts of these simulations. We clustered the resultant microstates (Supporting Information Figure S6) into six macrostates (Figure 6A) and then we generated and visualized representative coordinates from the two lowest energy states (see Figure 6B,C). It can be seen that the MSM captures a conformational transition from an α-helical structure for residues 60–70 to a conformation in which there is local loss of helicity, with the peptide chain folded back on itself. The reduced secondary structure and greater conformational flexibility in this region are in line with chemical shift-derived SSPs and ^15^N-relaxation data (Figure 4D and Supporting Information Figure S4).

This local structure with a bend around residue V66 (Figure 6C) is of potential interest because amyloid structures for αS determined by cryo-EM^91,92^ show a kernel structure near the dimer interface, which also has a bend around V66. Furthermore, biophysical studies of a peptide fragment αS(71–82) corresponding to close to this region demonstrate its propensity to form a β-sheet structure when bound to anionic lipid membranes. Our simulations thus suggest an attractive hypothesis (Figure 6D) in which a dynamic non-helical conformation formed on the bilayer surface could act as a potential seed for subsequent amyloid formation. Interestingly, a small molecule thought to interact with residues around 53–73 inhibits *in vitro* aggregation of αS.^93^

## Discussion and Conclusions

We have performed a multiscale MD simulation study along with biophysical measurements to explore the membrane-bound state(s) of αS. CG simulations, initiated from the structure of detergent micelle-bound αS as a representative folded state of the protein, suggest that major contacts between the protein and an anionic lipid bilayer involve the N-terminal helix and interhelical loop region. Recognizing the possible effects of restraints on the secondary structure within a CG simulation, we performed subsequent AT MD simulations, the analysis of which was aided *via* the construction of an MSM. These AT simulations (sampling conformational flexibility from three different CG-generated poses for membrane-bound αS) indicate the formation of a flexible non-helical region in the center of the initial helix 2 region, around residues 60–70; Figure 6D. This is in good agreement with NMR chemical shift data for αS bound to anionic phospholipid bicelles. Additionally, intramolecular contacts as identified by MD simulations were supported by chemical XLMS. The robustness of our findings is, thus, shown with a set of model membrane systems, a crucial finding given the high sensitivity of αS to the membrane system employed. MSMs based on the AT simulations revealed that the region around residues 60–70 can undergo a conformational transition to form a bend in the center of this region, which in turn correlates with a key bend in the proposed structure of amyloid fibrils formed by αS. Interestingly, recent solid state NMR data suggest that a peptide fragment close to this region of αS (residues 71–82) forms a β-sheet when bound to anionic lipid bilayers.^32^ Deep mutational scanning suggests formation of an extended membrane-bound α-helical conformation from residues 1–90 when αS is expressed in yeast, but these data are also consistent with increased dynamics in the C-terminal regions of this helix (*e.g.*, residues ∼70 onwards).^29^ Taking together these and related recent studies, our results suggest that by characterizing the interplay between the dynamic nature of membrane-bound αS (Figure 6D), its interactions with anionic lipids, and the role of such lipids (*e.g.*, PIPs) in cellular localization,^33^ we will eventually understand the relationship between the physical chemistry and biology of this complex membrane-binding protein.

## Supporting Information Available

CG simulations of the interaction of αS with a PG bilayer; CG simulations of the interactions with a PG membrane of αS models with various degrees of helicity; interaction of αS with a PC/PE/PS lipid layer; comparison of SSP scores calculated from NMR measurements using samples of αS bound to lipid bicelles, SDS micelles, or in aqueous solution; results of XLMS studies for αS when bound to POPG liposomes; and MSM of residues 60–70 (PDF)
