# Supplementary material for: Membrane Interactions of α-Synuclein Revealed by Multiscale Molecular Dynamics Simulations, Markov State Models, and NMR
Source: J Phys Chem B. 2021 Mar 15;125(11):2929–41. doi: 10.1021/acs.jpcb.1c01281 (PMC8006134; doi:10.1021/acs.jpcb.1c01281)
Supplement: Supplementary file 1 — jp1c01281_si_001.pdf [file jp1c01281_si_001.pdf]

Supporting Information for:

Membrane Interactions of α-Synuclein Revealed by Multiscale Molecular Dynamics Simulations, Markov State Models, and NMR

Sarah-Beth T. A. Amos[1], Thomas C. Schwarz[2], Jiye Shi[3], Benjamin P. Cossins[3], Terry S. Baker[3], Richard J. Taylor[3], Robert Konrat[2] and Mark S. P. Sansom[1]*

[1]Department of Biochemistry, University of Oxford, South Parks Road, Oxford OX1 3QU, UK

[2]Department of Structural and Computational Biology, Max Perutz Laboratories, University of Vienna, Campus Vienna Biocenter 5, A-1030 Vienna, Austria

[3]UCB Pharma, 208 Bath Road, Slough SL1 3WE, UK

*Correspondence: mark.sansom@bioch.ox.ac.uk

A

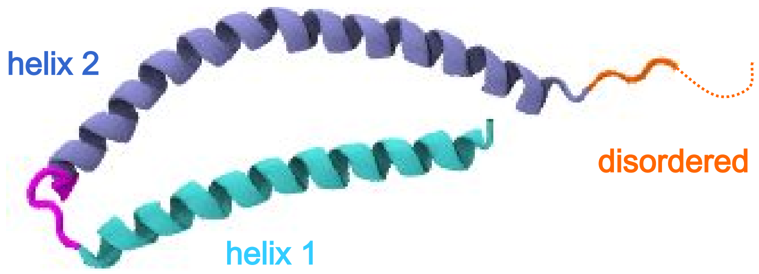

B

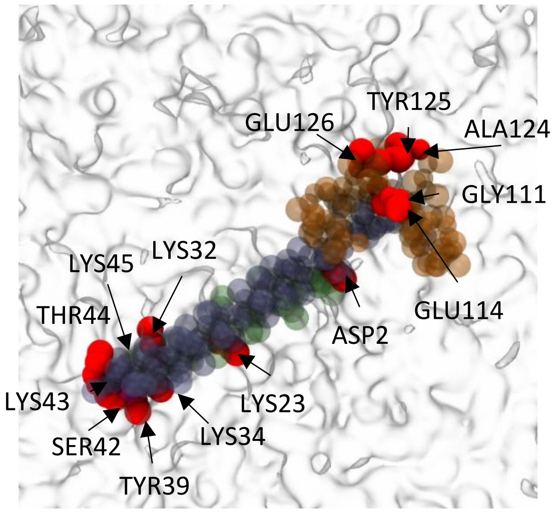

SI Figure S1:

Coarse grained simulations of the interaction of α-synuclein with a PG bilayer. **A** Initial structure (PDB id 1XQ8) for the CG simulations, with the region around residues 60-70 highlighted by the yellow circle (two helices 1 and 2 in cyan and grey/purple; interhelical loop in pink; C-terminal disordered region indicated by the orange broken line). **B** View from above of a representative PG-bound structure of α-synuclein with the top 14 residues making lipid contacts highlighted in red (see also Fig. 1E). PG headgroups are shown in grey.

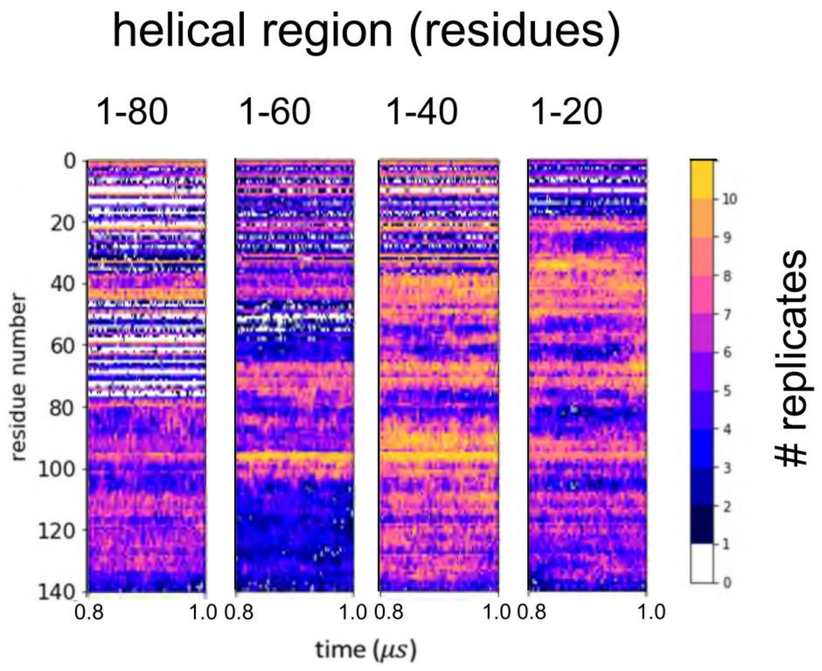

SI Figure S2:

Coarse grained simulations of the interactions with a PG membrane of α-synuclein models with various degrees of helicity. Each conformation was simulated for 10 replicates of 1 μs. The colours indicate the number of replicates across the ensemble making contacts at that time point. Only the last 0.2 μs of each set of simulations are shown. The contact profiles show that the contribution from the inter-helical region remains a common feature of binding, but abrogation of helicity results in a greater contribution from residues 45-100.

A

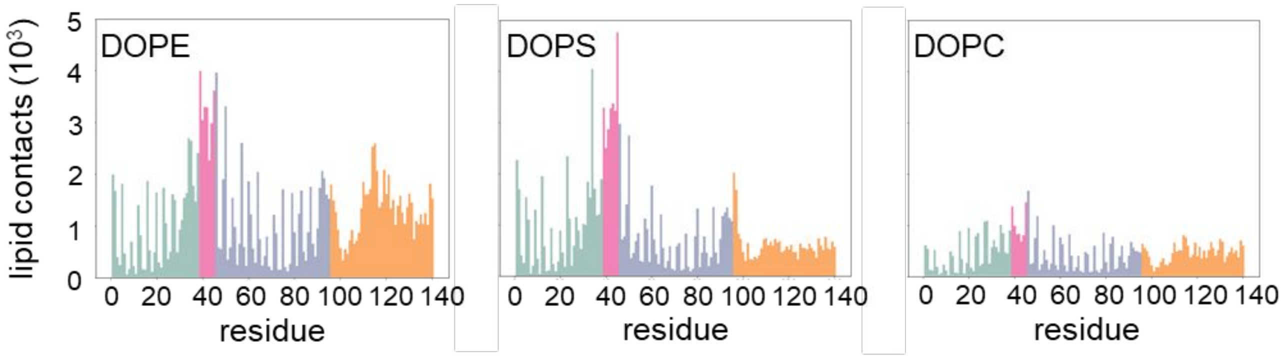

B

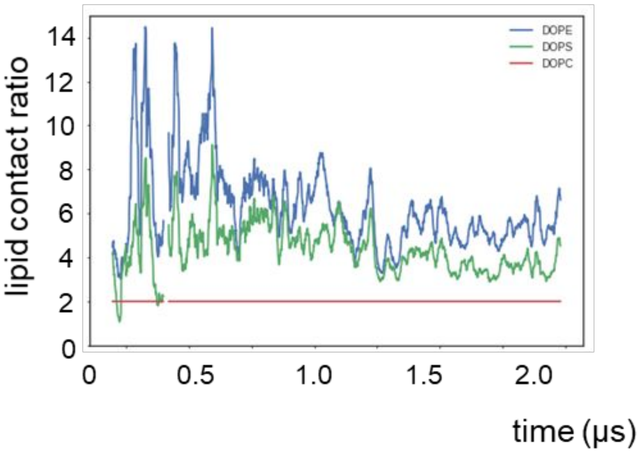

<u>*SI Figure S3:*</u>

Interaction of α-synuclein with a PC/PE/PS (2:5:3) lipid bilayer. Simulations were of 10 replicates each for 2 μs. **A** Lipid contacts shown separately for the three lipid species. Contacts for each residue of α-synuclein summed over the 10 simulation replicates. Colours on the histogram indicate the structural regions defined the main text (green = Helix 1, blue = Helix 2, pink = interhelical region, orange = disordered C-terminus). **B C**ontacts for each lipid species (PC = red; PE = blue; PS = green) shown as a function of time. These data correspond to those in main text Figure 2C replotted as ratios relative to PC (red line, set to 2 to reflect the bilayer composition ratio).

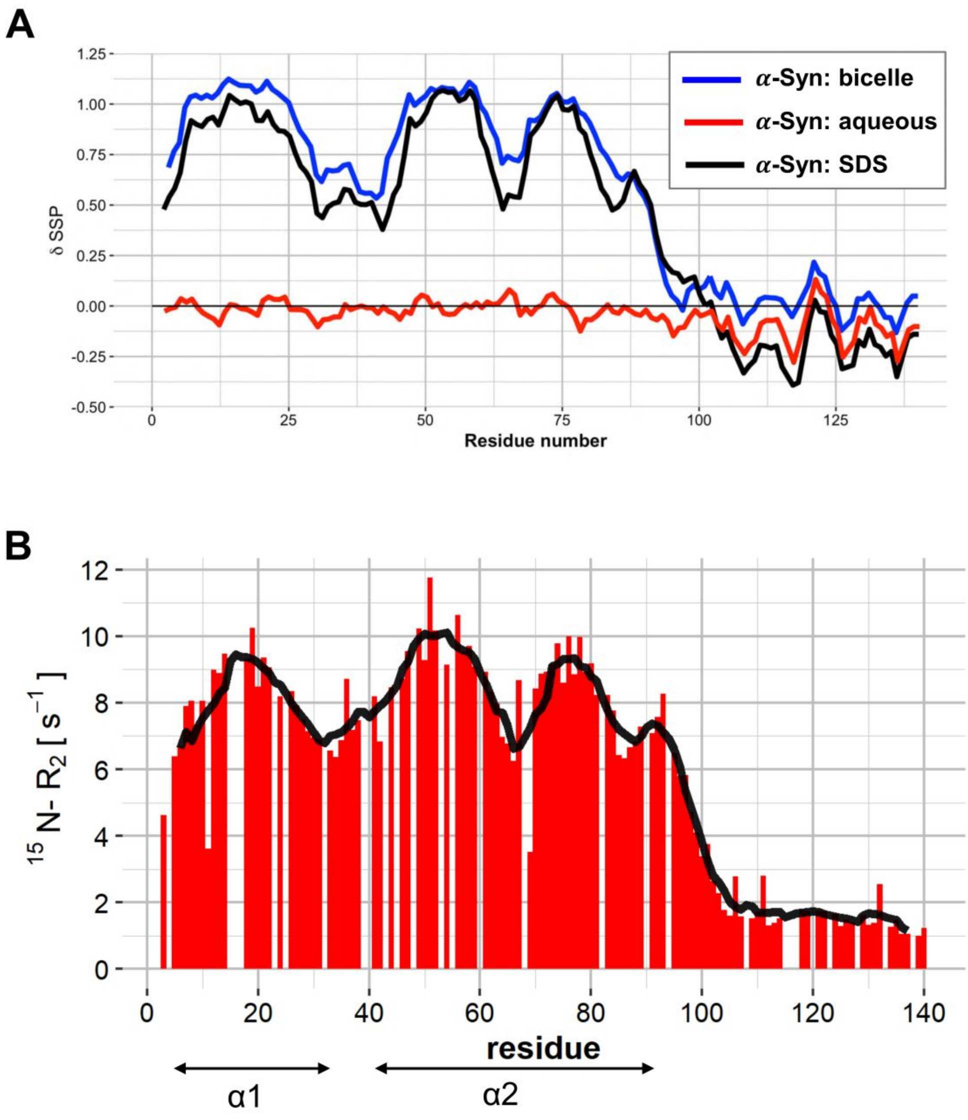

SI Figure S4:

A Comparison of secondary structure propensity (SSP) scores calculated from NMR measurements using samples of αS bound to lipid bicelles (blue; see main text, Figure 4C), to SDS micelles (black), or in aqueous solution (red). All were assigned at pH=5.5 and measured at 323 K. As described in the main text, secondary structure propensity values were calculated from Cα, Cβ, NH, N and CO values using the program SSP. The values obtained are the average of the shift observed versus random coil expected shifts, weighted by their sensitivity to α-helical or extended conformations. The observed Cα and Cβ shifts were also used for shift referencing, which removes the pH-dependent effects almost entirely. All three SSP score series were determined from αS samples recorded at the same buffer and temperature conditions. Assignments of the above conditions were carried out using standard-triple resonance experiments and include the used side-chain resonances, but do not include side-chain assignments. **B** Relaxation rates observed in SDS-bound αS. ^{15}N-R_2 rates of αS were measured in the same conditions as SSP-shifts of the same. The difference between micelle bound residues and the unbound C-terminus (98-140) is most clearly visible. Interestingly both the region between helices α1 and α2 (38-44) as well as within α2 (60-70) show slower relaxation as compared to tightly bound protein, indicating their higher flexibility.

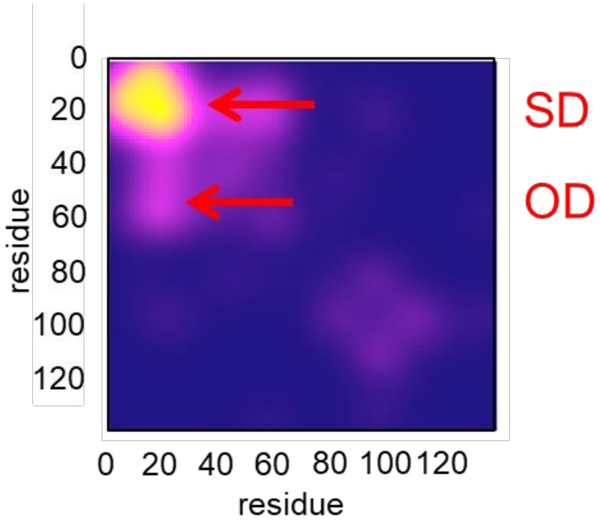

SI Figure S5:

Results of cross-linked mass spectrometry (XLMS) studies for αS when bound to POPG liposomes. The XLMS-data are used to determine a sum of all peptide spectrum matches (PSMs) found between two positions of the protein as in Figure 5A. The same data were used to calculate a density estimation of the XLMS crosslink-pattern, to simplify a visual comparison between the XLMS derived data and residue-residue distances generated from atomistic simulation ensembles (see Figure 5C). The red arrows indicate the strong diagonal (SD) and off-diagonal (OD) regions referenced in the main text.

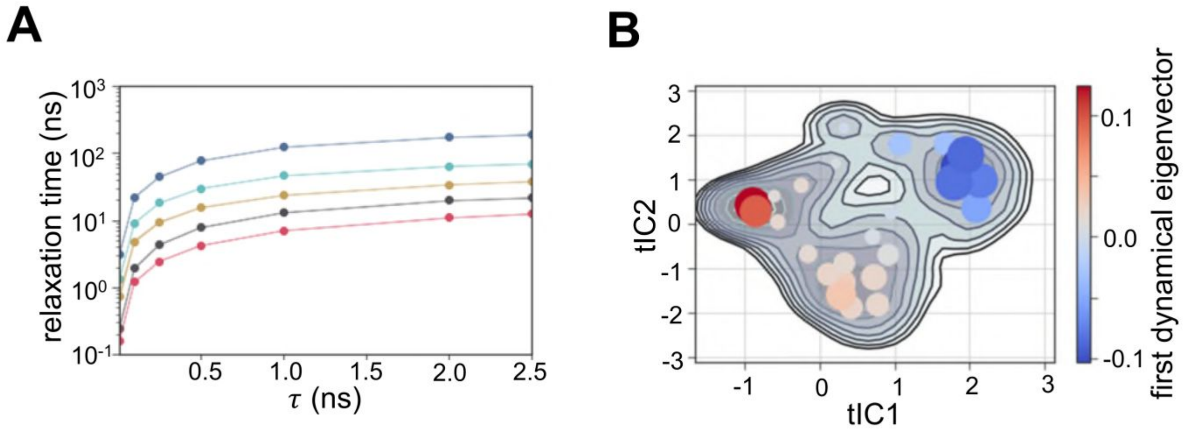

SI Figure S6:

Markov state model (MSM) of residues 60-70. As described in the main text, we used pooled data from the AT-MD simulations to construct an MSM featurised on the Cα contacts of these simulations. We plotted the implied timescales (**A**) to check for convergence selected an MSM lag time of 2 ns for model construction. **B** Slowest motion as represented by the first dynamical eigenvector from red states to blue. This corresponds to the slowest structural motion observed across the ensemble and can be interpreted as the major structural change.
